# Antitumor Effect of the Tyrosine Kinase Inhibitor Nilotinib on Gastrointestinal Stromal Tumor (GIST) and Imatinib-Resistant GIST Cells

**DOI:** 10.1371/journal.pone.0107613

**Published:** 2014-09-15

**Authors:** Hiroyuki Sako, Kazumasa Fukuda, Yoshiro Saikawa, Rieko Nakamura, Tsunehiro Takahashi, Norihito Wada, Hirohumi Kawakubo, Hiroya Takeuchi, Tai Ohmori, Yuko Kitagawa

**Affiliations:** Department of Surgery, School of Medicine, Keio University, Shinjuku-ku, Tokyo, Japan; University of Patras, Greece

## Abstract

Despite the benefits of imatinib for treating gastrointestinal stromal tumors (GIST), the prognosis for high risk GIST and imatinib-resistant (IR) GIST remains poor. The mechanisms of imatinib resistance have not yet been fully clarified. The aim of the study was to establish imatinib-resistant cell lines and investigate nilotinib, a second generation tyrosine kinase inhibitor (TKI), in preclinical models of GIST and imatinib-resistant GIST. For a model of imatinib-resistant GIST, we generated resistant cells from GK1C and GK3C cell lines by exposing them to imatinib for 6 months. The parent cell lines GK1C and GK3C showed imatinib sensitivity with IC_50_ of 4.59±0.97 µM and 11.15±1.48 µM, respectively. The imatinib-resistant cell lines GK1C-IR and GK3C-IR showed imatinib resistance with IC_50_ values of 11.74±0.17 µM (*P<0.001*) and 41.37±1.07 µM (*P<0.001*), respectively. The phosphorylation status of key cell signaling pathways, receptor tyrosine kinase KIT (CD117), platelet-derived growth factor receptor alpha (PDGFRA) and downstream signaling kinases: serine-threonine kinase Akt (AKT) and extracellular signal-regulated kinase 1/2 (ERK1/2) or the non-receptor tyrosine kinase: proto-oncogene tyrosine-protein kinase Src (SRC), was analyzed in established cell lines and ERK1/2 phosphorylation was found to be increased compared to the parental cells. Nilotinib demonstrated significant antitumor efficacy against GIST xenograft lines and imatinib-resistant GIST cell lines. Thus, nilotinib may have clinical potential for patients with GIST or imatinib-resistant GIST.

## Introduction

Gastrointestinal stromal tumor (GIST) is a mesenchymal cell neoplasm with an annual incidence of 10–20 cases per million people. While GIST rarely recurs locally after initial surgical resection, advanced disease is characterized by distant metastasis to the liver and serosal surfaces of the abdomen [1]. Approximately 20 years ago, the interstitial cells of Cajal (ICCs) were first identified as the cell of origin for GIST. Located between the autonomic nerves and the muscle walls of the gastrointestinal tract, ICCs are a population of cells that control peristaltic contractions and are characterized by their expression of the tyrosine kinase receptor KIT (CD117), a receptor for stem cell factor (SCF) [2]. SCF binding to KIT leads to receptor homodimerization and intracellular kinase activation, setting off a signaling cascade that positively regulates both the mitogen-activated protein kinase (MAPK) and phosphoinositide 3-kinase (PI3K)-AKT pathways [3]. The vast majority of GIST are positive for KIT protein expression, and 70–80% of GIST contain activating mutations in KIT, which result in constitutive activation via auto-phosphorylation and SCF-independent signaling and cellular proliferation [4].

Advances in the understanding of disease pathogenesis and molecular characteristics resulted in the use of novel tyrosine kinase inhibitors (TKIs), a class that includes imatinib (Novartis Pharma AG, Basel, Switzerland) and sunitinib (Pfizer Oncology, La Jolla, CA, USA) [3]. Although imatinib has been shown to improve survival in patients with GIST, drug resistance is inevitable for a majority of patients and presents a considerable clinical challenge [4]. Imatinib activity is limited by primary resistance to the drug in ∼15% of patients, and secondary resistance eventually develops in more than 80% of patients [5],[6]. Secondary resistance mainly occurs due to additional kinase domain mutations, which are thought to develop in viable tumor cells during imatinib therapy. However, the mechanisms that maintain the survival of these persistent cells after shutdown of KIT signaling by imatinib remain unclear.

The disease control time by imatinib is limited due to intolerance or resistance. Nilotinib (Tasigna, known as AMN107; Novartis Pharma AG) is a second-generation TKI that is expected to show enhanced clinical efficacy against GIST. Nilotinib is a potent TKI that has been shown both *in vitro* and *in vivo* to inhibit the auto-phosphorylation and proliferation of cells transformed with activating mutations of KIT or platelet-derived growth factor receptor-alpha (PDGFRA) tyrosine kinases, which are the kinases that are the key oncogenic drivers in GIST [7]–[9]. Imatinib resistance poses a significant challenge in the clinical management of GIST. The majority of approaches to date have involved the use of alternative small-molecule TKIs that target KIT or other receptor tyrosine kinases, such as PDGFRA and VEGFR. In the present study, we evaluated the antitumor effect of the tyrosine kinase inhibitor nilotinib for GIST and imatinib-resistant GIST.

Here we showed that nilotinib can potently inhibit the growth of GIST xenograft lines (GK1X, GK2X and GK3X) and cell lines (GK1C and GK3C) and imatinib-resistant GIST cell lines (GK1C-IR and GK3C-IR). Significantly, nilotinib effects on tumor growth were seen in both GIST and imatinib-resistant GIST, suggesting that treatment with nilotinib may represent a promising approach for overcoming the significant clinical challenge of imatinib resistance in GIST.

## Materials and Methods

### Chemicals

Imatinib mesylate (Product ID; CGP057148B) and nilotinib hydrochloride (product ID; AMN107-AAA.001) were provided by Novartis Pharma AG (Basel, Switzerland). For in vitro studies, a stock solution of 10 mM was prepared in 100% DMSO and then diluted to final concentration in medium. For peroral administration, the compound was dissolved in N-methyl-2-pyrrolidone (NMP) at 100 mg/mL and diluted 1∶10 (10 plus 90 v/v) with polyethylene glycol 300 (PEG300) to final 10 mg/mL.

### Cell culture

The human GIST cell lines GK1C and GK3C were established and characterized in our previous study [10]. The study was approved by the ethics committee of the chamber of surgeons of the School of Medicine, Keio University (no. 17–47). Human gastrointestinal stromal tumors (GISTs) were obtained from patients undergoing surgical resection following informed patient consent. The imatinib-resistant cell lines GK1C-IR and GK3C-IR were established from parent cell lines as imatinib-resistant cells that arose following continuous culturing in 5–10 µM imatinib for 6 months. All cell lines were cultured in RPMI-1640 supplemented with 10% heat inactivated fetal bovine serum (FBS; Invitrogen, Carlsbad, CA, USA) and 10,000 units/mL penicillin/streptomycin at 37°C and 5% CO_2_.

### GIST xenograft models and drug studies

All animal experiments were approved by the Laboratory Animals Center in Keio University, School of Medicine (Permit number: 09198). The GIST xenograft lines GK1X, GK2X and GK3X in nude mice were established from GIST patients as described in our previous study [10]. These xenograft lines were maintained by continual passage in BALB/cSLc-*nu/nu* mice. Mice bearing GK1X, GK2X and GK3X tumors (6–8 mice per group) were treated daily with vehicle or 40 mg/kg imatinib or nilotinib for 4 weeks. Tumor volume (TV) was determined from caliper measurements of tumor length (L) and width (w) according to the formula LW^2^/2. TV was determined every two to three days and on the day of evaluation. Mice were sacrificed and the percentage of tumor growth inhibition (TGI) was calculated as follows: TGI (%)  =  [1– (mean of treatment group tumor volume on evaluation day – mean of treatment group tumor volume on day 1)/(mean of control group tumor volume on evaluation day – mean of control group tumor volume on day 1)]×100.

### Proliferation assay

Cells were plated in 96-well microplates and cultured for 12 h before exposure to imatinib (1–100 µM) or nilotinib (1–100 µM) for 72 h. The cells were quantified by the WST-8 [2-(2-methoxy-4-nitrophenyl)-3-(4-nitrophenyl)-5-(2,4-disulfophenyl)-2H-tetrazolium, monosodium salt] assay as described by the manufacturer (Nacalai Tesque Inc., Kyoto Japan). The optical density (OD) was determined with Sunrise rainbow (Wako Pure Chemical Industries, Osaka Japan). The rate of inhibition was calculated as follows: % of inhibition  =  (OD of treated group – blank)/(OD of control group – blank) ×100%. The concentration of tested drugs resulting in 50% growth inhibition (IC50) was calculated.

### Immunostaining

GK1C-IR and GK3C-IR cell lines, and GK1X, GK2X and GK3X xenograft lines were stained by CD117 antibody (DAKO, Glostrup, Denmark) to assess KIT protein expression as described previously [10].

### Apoptosis assay

For an apoptosis assay, the supernatant was aspirated and cells were resuspended in 150 µL binding buffer, before staining with 5 µL Annexin V-FITC and 5 µL PI at room temperature for 25 min in the dark. After incubation, cells were processed as directed by the kit instructions (ANNEXIN V-FITC APOPTOSIS DETECTION KIT I, BD Pharmingen) and analyzed using a FITC signal detector and PI detector using a flow cytometer (FACSCalibur) and Cell Quest software (Becton Dickinson).

### Flow cytometry analysis

Cells were washed in PBS and fixed with 2% paraformaldehyde (PFA) in a 37°C water bath for 10 min. Then, cells were washed with PBS and pelleted by centrifugation (800×g) for 5 min, and the supernatant was removed. Cells were mixed to disrupt the pellet and permeabilized by adding 500 µL 90% methanol (for 1–10×10^6^ cells) and incubated on ice for 15 min. After blocking on ice for 10 min, cells were then washed and incubated with primary antibodies against phospho-KIT (Tyr719), phospho-PDGFRA (Tyr754), phospho-Src (Tyr416), phospho-AKT (Ser473), and phospho-ERK1/2 (Thr202/Tyr204) (Cell Signaling Technology, Inc., Danvers, MA) for 30 min at room temperature. Cells were washed with PBS before incubation for 30 min with Alexa Fluor 488 donkey anti-rabbit IgG antibody (Life Technologies, Carlsbad, CA). Then, each sample was analyzed using a FACSCalibur (Becton Dickinson). Cell distribution was analyzed using FlowJo software (TomyDigital Biology).

### Statistical analysis

Data values are expressed as means ± SD or mean-fold change. The statistical significances of mean values were determined by Student's t-test. P-value ≤0.05 was considered significant for the Student's t-test.

## Results

### Antitumor effect for GIST xenograft lines

**Figure 1:**
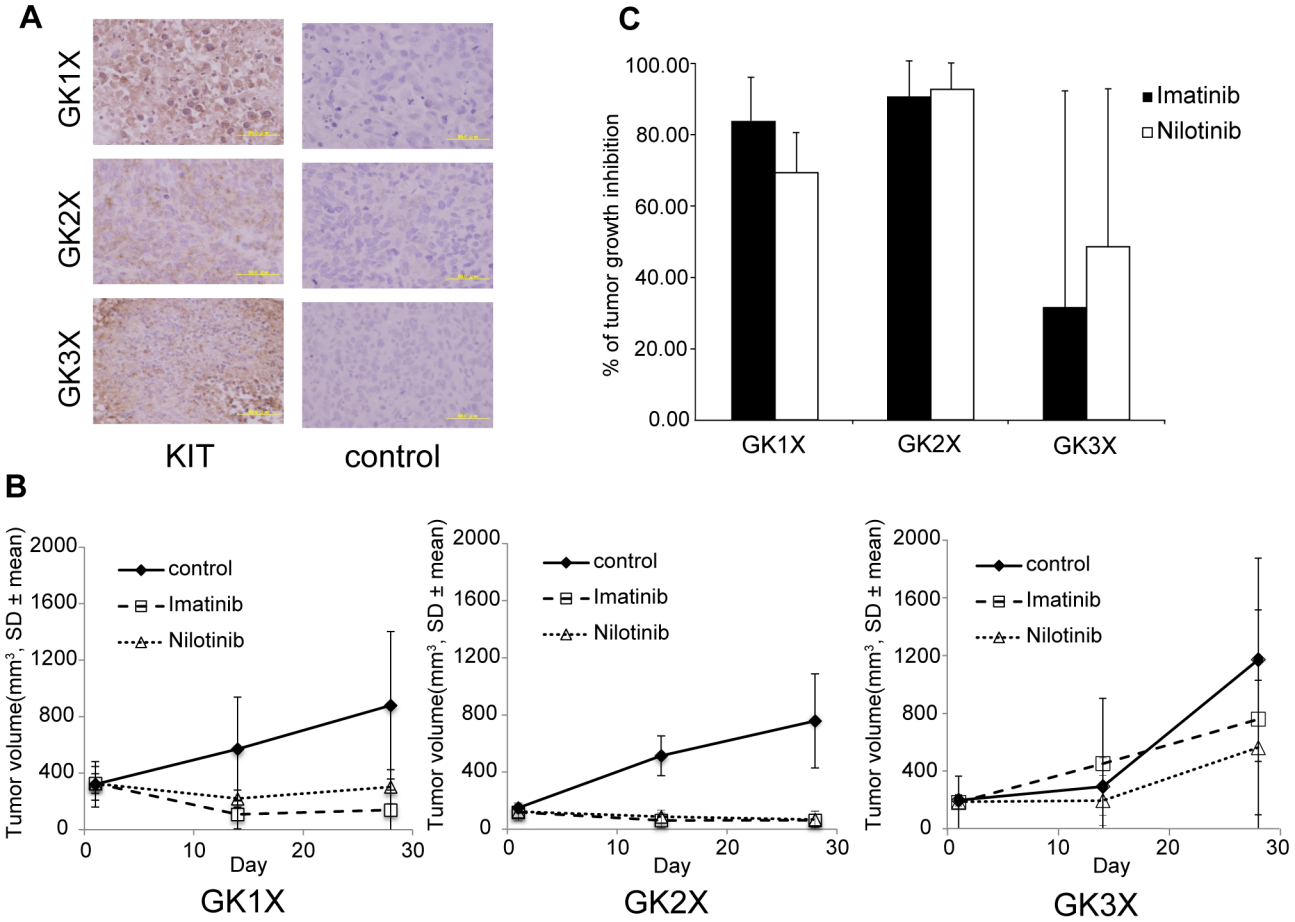
Nilotinib antitumor activity in GIST xenograft models. (A) Immunohistochemistry staining for KIT in xenograft lines established from human GISTs: GK1X, GK2X and GK3X. (B) Tumor tissue fragments (∼5 mm^3^) were transplanted s.c. into the backs of BALB/cSlc-*nu/nu* mice that were randomized into 3 groups (n = 6–8). Doses of 40 mg/kg/day of imatinib, nilotinib or pure water (control) were administered by oral gavage daily for 28 days. Tumor size was measured every two to three days. (C) Tumor growth inhibition (TGI) on the day of evaluation was calculated as the ratio of tumor volume on the evaluation day to that on day 1.

To determine whether nilotinib therapy would have benefits that are not inferior to imatinib, we demonstrated the efficacy of antitumor activity in KIT-positive GIST xenograft lines (GK1X, GK2X and GK3X) (Fig. 1A). Tumor volume (TV) was evaluated between groups every two to three days. Fig. 1B shows the TV change in each group. The percentage of tumor growth inhibition (TGI) was 83.8% for imatinib and 69.6% for nilotinib in the GK1X xenograft line (n.s.). In the GK2X xenograft line, TGI was 83.0% for imatinib and 85.3% for nilotinib (n.s.). Additionally, the GK3X xenograft line TGI was 31.1% for imatinib and 47.5% for nilotinib (n.s.) (Fig. 1C). These results suggested that, except for the GK1X xenograft line, nilotinib showed equivalent or higher antitumor effects than imatinib.

### Establishment of imatinib-resistant GIST cell lines

**Figure 2:**
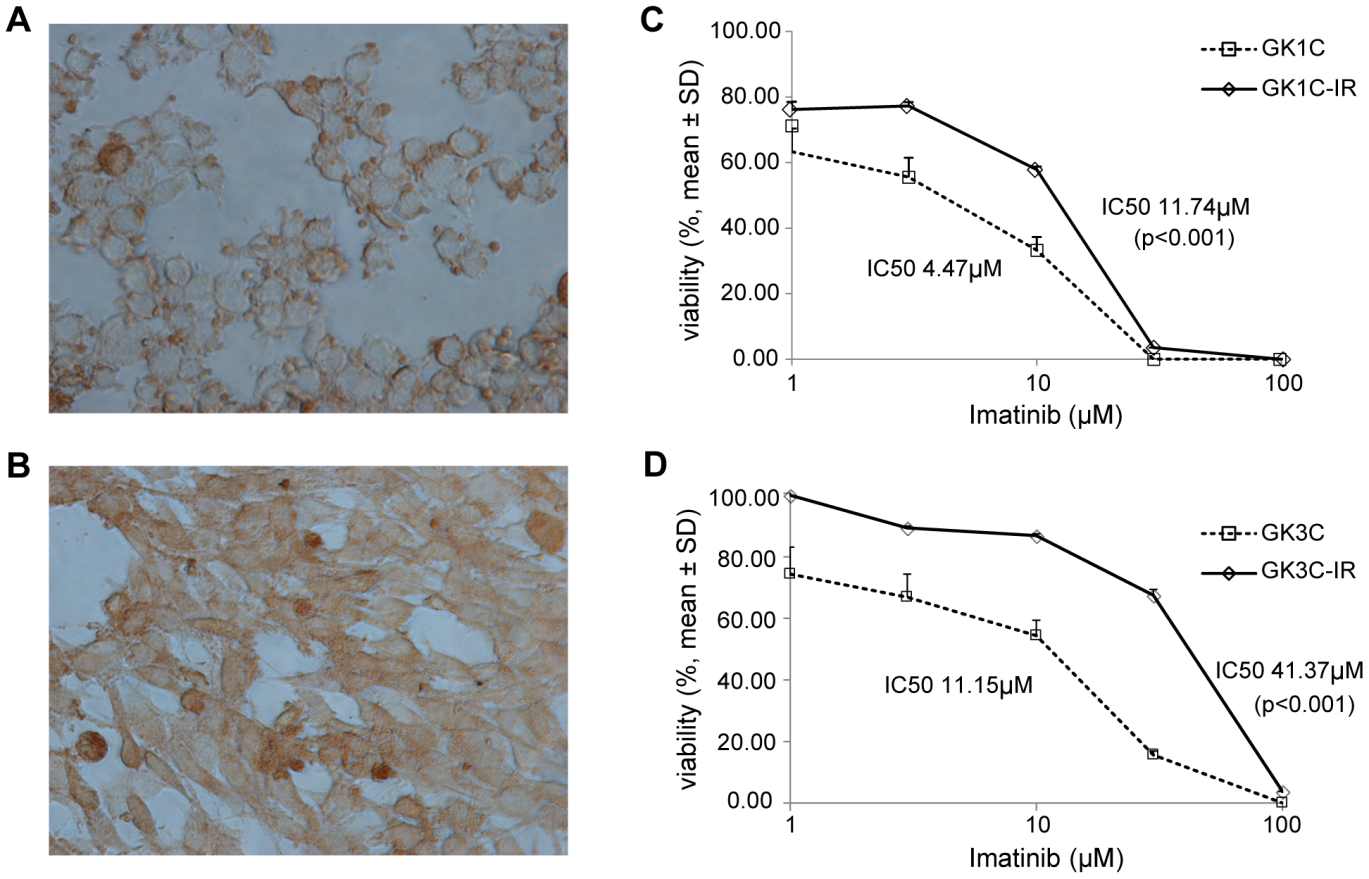
Establishment of imatinib-resistant GIST cell lines. (A, B) Immunohistochemical assay of KIT expression in GK1C-IR and GK3C-IR as determined by staining with DAB (magnification, 400x). (C, D) GK1C and GK1C-IR cells, GK3C and GK3C-IR cells, 2.5×10^3^ cells (per well) were seeded into 96-well microplates in triplicate 12 h before treatment, and then exposed to different concentrations (0∼100 µM) of imatinib for 72 h. The percentage of cellular proliferation was gauged using the WST-8 method. Imatinib-resistant (IR) cells showed resistance to imatinib with IC_50_ of 11.74±0.17 µM (*p<0.001*) or 41.37±1.07 µM (*p<0.001*). Data are presented as means ± SD and evaluated using Student's *t* test.

**Figure 3:**
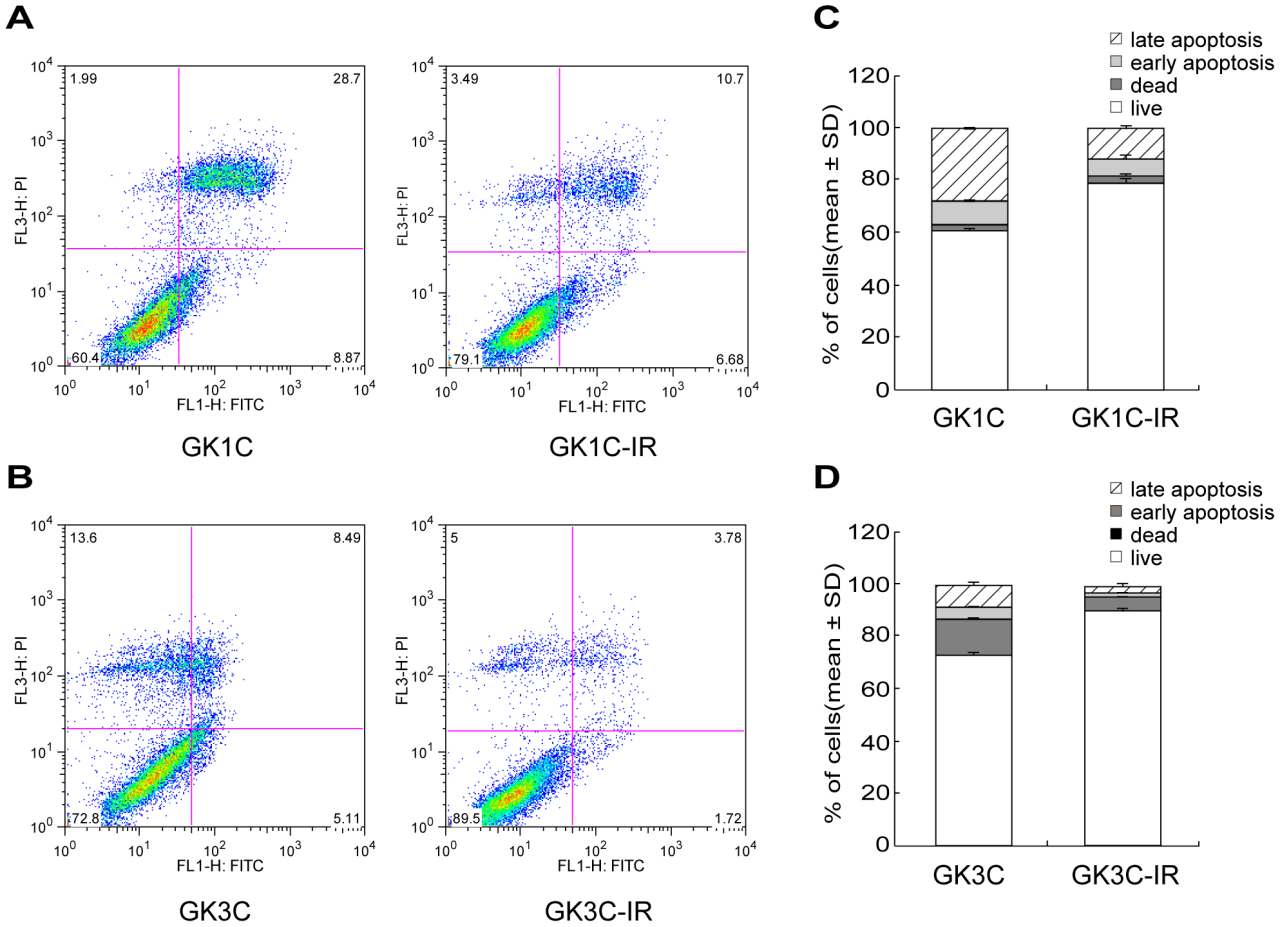
Apoptosis assay. The effects of imatinib on apoptosis were evaluated using a FITC Annexin V apoptosis detection kit (Becton Dickinson). Cells were then treated with doses of imatinib IC_50_ concentration (4.47 µM for GK1C and GK1C-IR, 11.15 µM for GK3C and GK3C-IR). For the apoptosis assay, the supernatant was aspirated, and cells were then resuspended in 150 µL of binding buffer, and stained with 5 µL Annexin V-FITC and 5 µL propidium iodide at room temperature for 5 min. After incubation, cells were processed as directed by the manufacturer and analyzed using a FITC signal detector and propidium iodide detector using a BD FACScaliburTM device. (A, C) Parent cells: Live 60.2±1.2%, Dead/apoptotic 39.1± 0.5%, IR: Live 78.7±1.6%, Dead/apoptotic 21.0±1.9% (*P<0.01*), (B, D) Parent cells: Live 72.8±0.1%, Dead/apoptotic 27.2±0.9%, IR: Live 90.0±0.5%, Dead/apoptotic 10.0±0.5% (*P<0.01*).

To investigate mechanisms of imatinib-resistant GIST, we generated resistant cells from GK1C and GK3C cells by exposing them to imatinib for 6 months. CD117 (KIT) protein expression was determined by immunocytochemistry on the human GIST cell lines GK1C-IR and GK3C-IR (Fig. 2A, B). The parental cell lines GK1C and GK3C showed imatinib sensitivity with IC_50_ of 4.47±0.97 µM and 11.15±1.48 µM, respectively. In contrast, the resulting cell lines GK1C-IR and GK3C-IR demonstrated *in vitro* imatinib resistance with IC_50_ of 11.74±0.17 µM (*P<0.001*) and 41.37±1.07 µM (*P<0.001*), respectively (Fig. 2C, D), which represent increases of 2.6 and 3.7 relative to the respective parental cell lines. Additionally, to analyze the mechanism underlying the growth inhibition induced by imatinib, we examined the effects of imatinib on apoptotic cell death. For this purpose, we used flow cytometry to determine the percentage of Annexin V-positive cells among imatinib-resistant and parent cells treated with imatinib. The results of this assay demonstrated that imatinib-resistant GIST cell lines (GK1C-IR and GK3C-IR) showed a significant decrease in the induction of apoptosis with imatinib as compared to parent cells (*P<0.01*) (Fig. 3).

### Profiles of cellular protein phosphorylation

**Figure 4:**
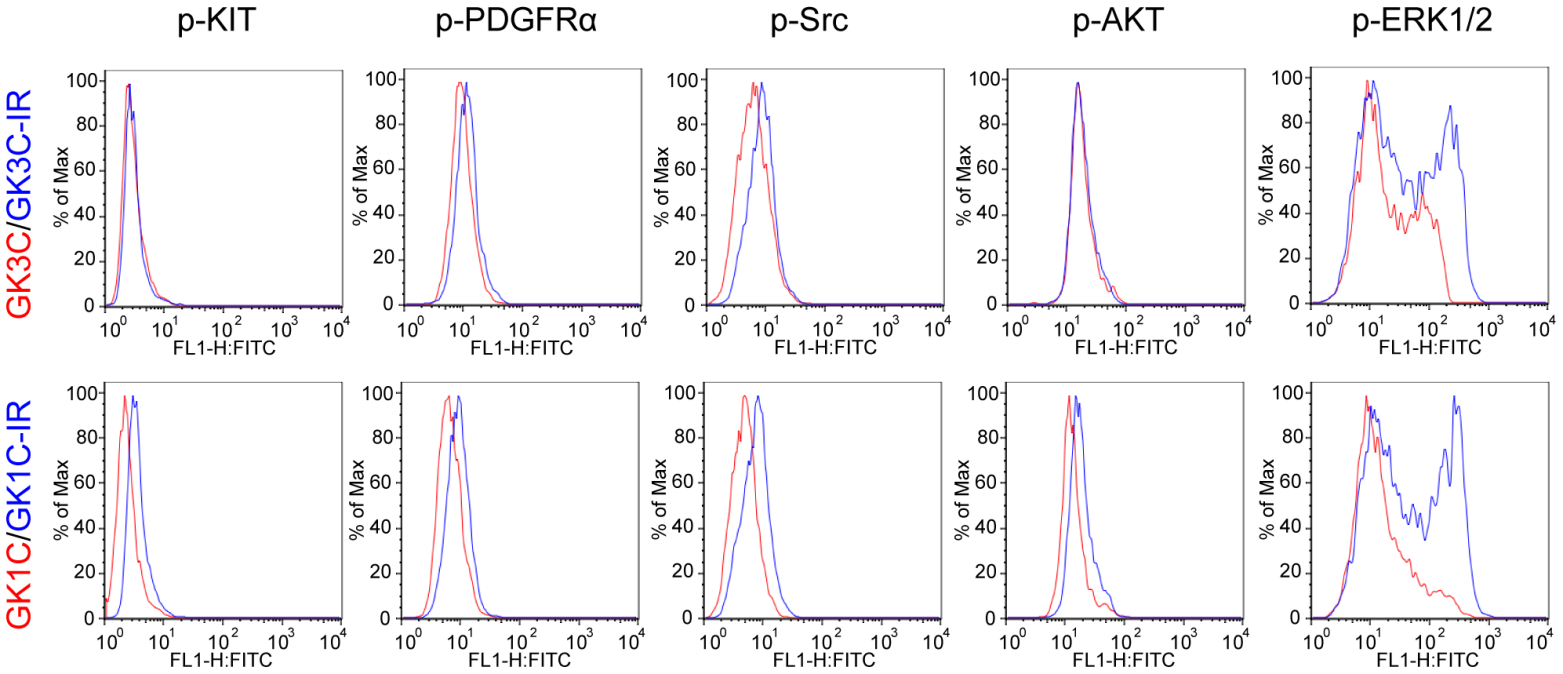
Quantitative phosphorylation analysis. Parental (GK1C and GK3C; red histograms) or imatinib-resistant GIST cell lines (GK1C-IR and GK3C-IR, blue histograms) were fixed and stained with anti phospho-KIT (Tyr719), anti phospho-PDGFRA (Tyr754), anti phospho-SRC (Tyr416), anti phospho-AKT (Ser473) and anti phospho-ERK1/2 (Thr202/Tyr204). Finally, cells were detected with Alexa Fluor 488 donkey anti-rabbit IgG antibody (Isotype control was reacted only with the secondary antibody). The MFI (mean of fluorescence intensity) values were calculated by FlowJo. GK1C: p-KIT = 3.21, p-PDGFRA = 10.3, p-SRC = 7.19, p-AKT = 20.3, p-ERK1/2 = 37.8. GK1C-IR: p-KIT = 3.30, p-PDGFRA = 12.8, p-SRC = 9.35, p-AKT = 20.5, p-ERK1/2 = 94.4. GK3C: p-KIT = 2.65, p-PDGFRA = 7.29, p-SRC = 5.35, p-AKT = 19.5, p-ERK1/2 = 32.2. GK3C-IR: p-KIT = 3.89, p-PDGFRA = 9.82, p-SRC = 8.31, p-AKT = 21.3, p-ERK1/2 = 115.

The profiles of the main cell signaling activity of phospho-KIT (Tyr719), phospho-PDGFRA (Tyr754), phospho-SRC (Tyr416), phospho-AKT (Ser473) and phospho-ERK1/2 (Thr202/Tyr204) were analyzed by flow cytometry in GK1C-IR and GK3C-IR cells and compared to that of the parent cells. The phosphorylation status was detected as the mean of fluorescence intensity (MFI) by analysis software (FlowJo). Interestingly, there was little difference in the phosphorylation state of KIT, PDGFRA, AKT and SRC in the parent and imatinib-resistant cells. In contrast, the phosphorylation of ERK1/2 showed about a 2.50 and 3.57 fold increase in resistant cells, respectively (Fig. 4), which could provide an advantage for survival and proliferation.

### Efficacy of nilotinib for imatinib-resistant GIST cell lines

**Figure 5:**
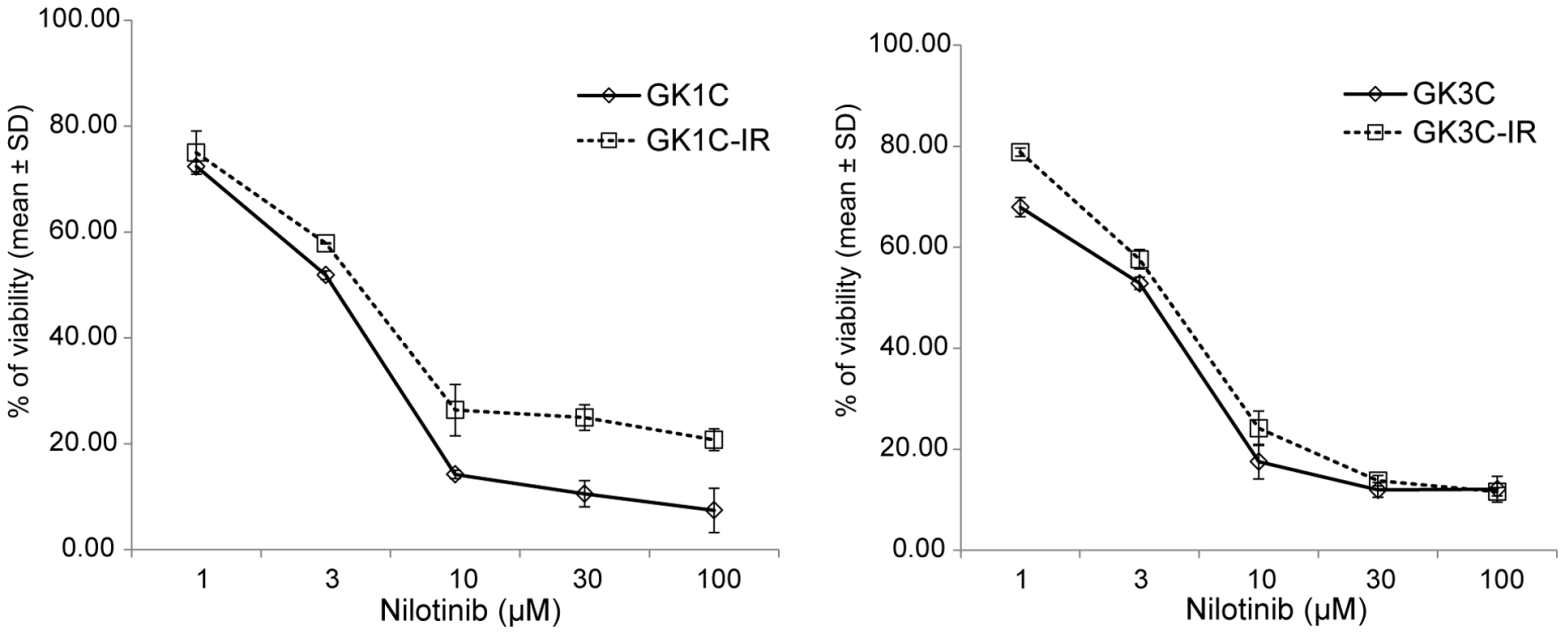
Antitumor activity of nilotinib on GIST and imatinib-resistant GIST cells. Cells were maintained in supplemented medium for 12 h, and then incubated with nilotinib (0∼100 µM) for 72 h. Cell viability was determined by comparing treated cells with the untreated control. Data are means of triplicates from a representative experiment.

We evaluated whether the tyrosine kinase inhibitor nilotinib could inhibit the proliferation of parent GIST cell lines (GK1C and GK3C) and imatinib-resistant GIST cell lines (GK1C-IR and GK3C-IR). Parent and imatinib-resistant GIST cell lines showed sensitivity to nilotinib in a concentration-dependent manner (Fig. 5) with the IC_50_ values of parent GIST cell lines being 3.15±0.31 µM for GK1C and 3.32±0.18 µM for GK3C (n.s.), and the imatinib-resistant cell lines showing IC_50_ values of 4.10±0.46 µM and 3.96±0.19 µM for GK1C-IR and GK3C-IR (n.s.), respectively. These results indicate that nilotinib inhibited the proliferation of both imatinib-resistant and parent GIST cell lines, with IC50 values not varying significantly between the individual cell lines.

## Discussion

Gastrointestinal stromal tumor (GIST) is the most common mesenchymal malignancy of the gastrointestinal tract. Prior to the availability of imatinib mesylate (imatinib), the prognosis of some advanced patients with unresectable or recurrent GIST was poor [11]. Recently, imatinib has become the standard therapy for recurrent and metastatic GIST [12]–[14]. Two large, randomized control phase III trials reported the activity and efficacy of imatinib in advanced GIST patients, both in terms of progression-free survival (PFS) and overall survival (OS) [15],[16]. The major limitation of such a highly effective therapy has been the development of secondary resistance. Primary resistance refers to patients who achieve no response. In the past few years, several TKIs, such as dasatinib, sorafenib, regorafenib and pazopanib were investigated for their effect on imatinib-resistant GIST [17]–[23]. On the other hand, nilotinib was designed based on the structure of imatinib and shows higher affinity for the ATP- binding site of ABL kinase. Nilotinib can overcome imatinib-resistant chronic myeloid leukemia (CML) and also selectively inhibits KIT and PDGFR [24]. At the GIST, there is a report to make that nilotinib worked to imatinib tolerance GIST [25],[26]. We previously established cell lines (GK1C and GK3C) and xenograft lines (GK1X, GK2X and GK3X) from GIST patients, and our GIST model were capable of repeated passage for long periods, which made them useful for the study of GIST [10]. In this study, we showed that treatment of GIST xenograft lines with nilotinib resulted in a decrease in tumor growth, suggesting that nilotinib-mediated growth inhibition in GIST might be occurring through interference with the activity of KIT or PDGFRA signaling pathways. On the other hand, imatinib showed a sufficient antitumor effect, but it has a shorter plasma half-life in mice than nilotinib. Therefore, these doses may not provide equivalent drug exposure to the mice.

Resistance to chemotherapy remains a major obstacle for the treatment of GIST. As such, understanding the molecular mechanism of resistance is important to the development of effective new therapies for treatment of this disease. The effect of imatinib is different for various types of *c-kit* and *PDGFRA* mutations and secondary resistance against imatinib is often acquired by a secondary mutation or amplification of *c-kit* or *PDGFRA* expression [27]–[30]. However, only about half of GIST with secondary resistance to imatinib has secondary mutations in *c-kit* or *PDGFRA* [31]–[33] and in many cases, the efficacy of imatinib has not depended on the pattern of *c-kit* or *PDGFRA* expression. As a cause of other secondary resistance, there are a report to be due to the overexpression of drug efflux pump such as MDR1, the report to the low activity of OCT-1 in connection with the intracellular taking-in of imatinib [34], the report to make due to low PTEN protein expression and activation of PI3K/AKT and MAPK pathway [35], SCF which is the ligand of KIT is activated by the administration of imatinib and is affecting the acquisition of tolerance [36]. Recently, several studies reported that a switch of protein kinase phosphorylation that occurs in secondary resistance of GIST is a key event in tumor cell survival and proliferation [37],[38]. To explore the alteration of phosphorylation levels of tyrosine kinase induced by imatinib, we generated resistant cells from imatinib-sensitive GK1C and GK3C cells by exposing them to increasing concentrations of imatinib for 6 months. The resultant cell lines GK1C-IR and GK3C-IR showed resistance to imatinib *in vitro* with IC_50_ of 11.74±0.17 µM and 41.37±1.07 µM, respectively. In an apoptosis assay, these imatinib-resistant cells (GK1C-IR and GK3C-IR) showed a decrease in the size of dead/apoptotic populations. Unlike in the parent cells, phosphorylation of KIT or PDGFRA or its downstream intermediates such as AKT, and the non-receptor tyrosine kinase Src in imatinib-resistant GIST cell lines remained unchanged even in the presence of imatinib. Moreover, phosphorylation ERK1/2 was accelerated following imatinib treatment. While patients with GIST usually respond to imatinib, most eventually experience disease progression with the reactivation of KIT tyrosine kinase and its downstream signaling pathways [31],[39]. On the other hand, the BFR14 trial showed that stopping imatinib treatment, even after a complete response, resulted in disease progression or recurrence [40],[41]. Treatment of chronic myelogenous leukemia (CML) indicated that secondary mutations might emerge after imatinib therapy [42]. Thus, imatinib resistance may occur through an acquired add-on secondary mutation in the ATP binding domain or the activation-loop domain of KIT, KIT overexpression and/or activation of alternative cell signaling pathways [33].

In the past few years, several tyrosine kinase inhibitors (TKIs), including nilotinib, dasatinib and sorafenib were investigated for use in treating imatinib- or sunitinib-resistant GIST. We evaluated the inhibitory efficacy of nilotinib against GIST xenograft lines (GK1X, GK2X and GK3X) and imatinib-resistant GIST cell lines (GK1C-IR and GK3C-IR). These survival assays demonstrated that nilotinib inhibited the proliferation of both the xenograft lines and imatinib-resistant cell lines. Moerover, to investigate the antitumor effect of nilotinib in vivo, we have tried for imatinib-resistant GIST xenografts. Imatinib resistant GIST cell lines (GK1C-IR and GK3C-IR) were prepared and expanded, and 2×10^6^ cells were transplanted into the back of SCID (C.B-17/IcrHsd-Prkdc scid) mice (each 10 mice). However, cells did not grow in mice, we could not acquire imatinib-resistant GIST xenografts. In many other studies, it has not yet been reported for imatinib resistant GIST cell lines in vivo drug assay [33],[37],[38]. Future, the establishment of in vivo model and validation of the antitumor effect of nilotinib are required.

ENESTg1 (evaluating Nilotinib Efficacy and Safety in Clinical Trials Versus Imatinib in Adult Patients With Unresectable and/or Metastatic GIST) Phase III study, the clinical utility of nilotinib did not demonstrate the superiority of Tasigna over imatinib. On the basis of the trial results, nilotinib is not generally recommended for GIST therapy. Nevertheless, this new TKI may merit to patients with high risk GIST or imatinib-resistant GIST.
